# Gastric functional monitoring by gastric electrical impedance tomography (*g*EIT) suit with dual-step fuzzy clustering

**DOI:** 10.1038/s41598-022-27060-7

**Published:** 2023-01-10

**Authors:** K. Sakai, P. N. Darma, P. A. Sejati, R. Wicaksono, H. Hayashi, M. Takei

**Affiliations:** 1 Department of Mechanical Engineering, Graduate School of Science and Engineering, Chiba University, Chiba-Shi, Japan; 2 Division of Fundamental Engineering, Department of Mechanical Engineering, Graduate School of Science and Engineering, Chiba University JSPS International Research Fellow, Chiba-Shi, 263-8522 Japan; 3 Center for Frontier Medical Engineering, Chiba University, Chiba-Shi, Japan

**Keywords:** Computational biophysics, Biomedical engineering, Electrical and electronic engineering

## Abstract

Gastric Function has been successfully estimated by gastric electrical impedance tomography (*g*EIT) Suit with dual-step fuzzy clustering. The *g*EIT Suit which are made of elastic cloth with dual-planar electrodes and compact data acquisition (DAQ) system measures gastric impedance Z to visualize the gastric conductivity distribution σ. The dual-step fuzzy clustering extracts the clustered gastric conductivity distribution ^*k*^σ, which accurately estimates the gastric function*.* The *g*EIT Suit with dual-step fuzzy clustering are applied to eight healthy persons during liquid meal consumption to estimate the gastric function under gastric accommodation phase of 200, 400 and 600 mL based on the gastric emptying phase. As the results, the *g*EIT Suit successfully estimate the gastric function. By the measured impedance Z, the subjects have a mean temporal impedance $\overline{\Delta \mathbf{Z} }$= − 9.27 [Ohm] and *p*-value of that $\overline{\mathbf{Z} }$ *p*(*Z*) = 0.0013[–]as the t-test result. In the case of gastric conductivity distribution σ, the subjects have a value of spatial mean conductivity distribution ⟨σ⟩ = 0.23[–] and *p*-value of that ⟨σ⟩ *p*(σ) = 0.0140[–]. Lastly, in the case gastric volume *V*, subjects have a gastric volume *V* = 12.44 [%] and *p*-value *p*(*V*) = 0.0664[–].

## Introduction

Monitoring of gastric function by measuring^1^ is a critical parameter in many clinical conditions such as aspiration of gastric content during anesthesia^2^, gastroesophageal reflux diseases^3^, gastroparesis diseases^4^, and functional dyspepsia diseases^5^ to estimate gastric function. Proper daily monitoring of gastric function is necessary to improve the patient’s quality of life. Several methods are currently used to monitor gastric function, which are Electro Gastro Graphy (EGG), intra-gastric barostat (IGB), dynamic magnetic resonance imaging (MRI). The EGG is the noninvasive technique of elctro physological used to record gastric function by electrical activity measured from electrodes placed on the surface of abdomen as a representation of the gastric function^6^. The Electrical Impedance Tomography is also the noninvasive and electrical technique to evaluate gastric function. The intra gastric barostat is considered as the golden standard to directly measure gastric function using polyethylene bag implanted in the proximal stomach through inflation or deflation of the polyethylene bag. During emptying phase, the intra gastric barostat detects decreasing of polyethylene bag volume as the results of increasing pressure in the proximal stomach^7^. MRI determines gastric function level using interaction of magnetic field and particle properties inside stomach, then simultaneous MRI scans the abdomen to determine gastric functional activity through MRI volumetric image reconstruction^8^. On the other hand, ultrasound determines gastric function level using reaction of emitted ultrasound, then ultrasound scans on the abdomen to determine the gastric function through combination of several number of 2D images through movement of ultrasound transducer array on the stomach^9^. Nonetheless, these current standard methods have some drawbacks which are invasive, radioactive, expensive and massive which are impractical for daily monitoring of gastric function.

In order to solve problem that there is no daily gastric function measurement method, because conventional techniques are not suitable: expensive, large, invasive, for monitoring gastric function, a recent development in electrical impedance tomography (EIT)^10^ and mobile data acquisition systems^11^ has opened the possibility of daily gastric morphological imaging. EIT has been successfully applied as an alternative bio-medical morphological imaging such as functional lung imaging^12^, muscle imaging^13^, and breast cancer imaging^14^, abdomen subcutaneous fat imaging^15^, and urinary bladder imaging^16^.

Although EIT is able to image human body parts such as the lung, muscle, breast, subcutaneous abdominal fat and urinary bladder, it is extremely difficult to apply EIT to human gastric functional monitoring, since the human stomach is located near to multiple layers of organs which have similar electrical properties, such as the kidney, liver, skin, spinal cord and spleen, as shown in Table 1^17^,that conductivity are only 1/4 or more smaller than gastric conductivity. Those abdomen organs surrounded gastric make gastric conductivity distribution image by EIT noisy.

**Table 1:** Dielectric properties of human abdomen tissues ^17^.

Tissue	Conductivity [S/m]	Permittivity [–]
Fat	0.042	911.5
Kidney	0.137	38748.0
Liver	0.053	28
Skin	0.0002	1133.5
Spinal cord	0.004	35568.9
Spleen	0.110	13891.2
Gastric	0.530	8698.7

In order to resolve the difficulties, mitigate influence from gastric surrounded organs and clear noise from EIT image, we already proposed gastric electrical impedance tomography (*g*EIT) method by 3D full Jacobian matrix **J** and dual-step fuzzy clustering for extracting gastric function in-vitro experiments during gastric empty phases, fundus relaxation phase, corpus relaxation phase and antrum relaxation phase^18^.

However, our previous *g*EIT method has several drawbacks to apply to a real gastric human imaging such as (1) the static *g*EIT sensors were used in the experimental study using typical abdomen geometry with the electrodes were placed in the rigid position. On the other hand, the electrodes should be placed around an abdomen segment with a flexible shape and different sizes in gastric function monitoring. (2) the huge and heavy impedance analyzer was used to measure the impedance during the experimental study. On the other hand, measurement equipment should be smaller for easy using as daily gastric function monitoring.

In this paper, a non-invasive *g*EIT Suit is applied to estimate gastric function in eight healthy subjects during gastric emptying phase through overnight fasting and gastric accommodation phase. The proposed method has 2 stages which are (1) fabrication of *g*EIT Suit by integration of elastic suit with dual-planar EIT electrode arrangement with compact data acquisition (DAQ) system to measure the impedance **Z**, and (2) reconstruction of gastric conductivity distribution **σ** with dual step fuzzy clustering estimate the gastric volume *V*.

## Gastric EIT (*g*EIT) suit with dual-step fuzzy clustering

### Fabrication of *g*EIT suit

**Figure 1:**
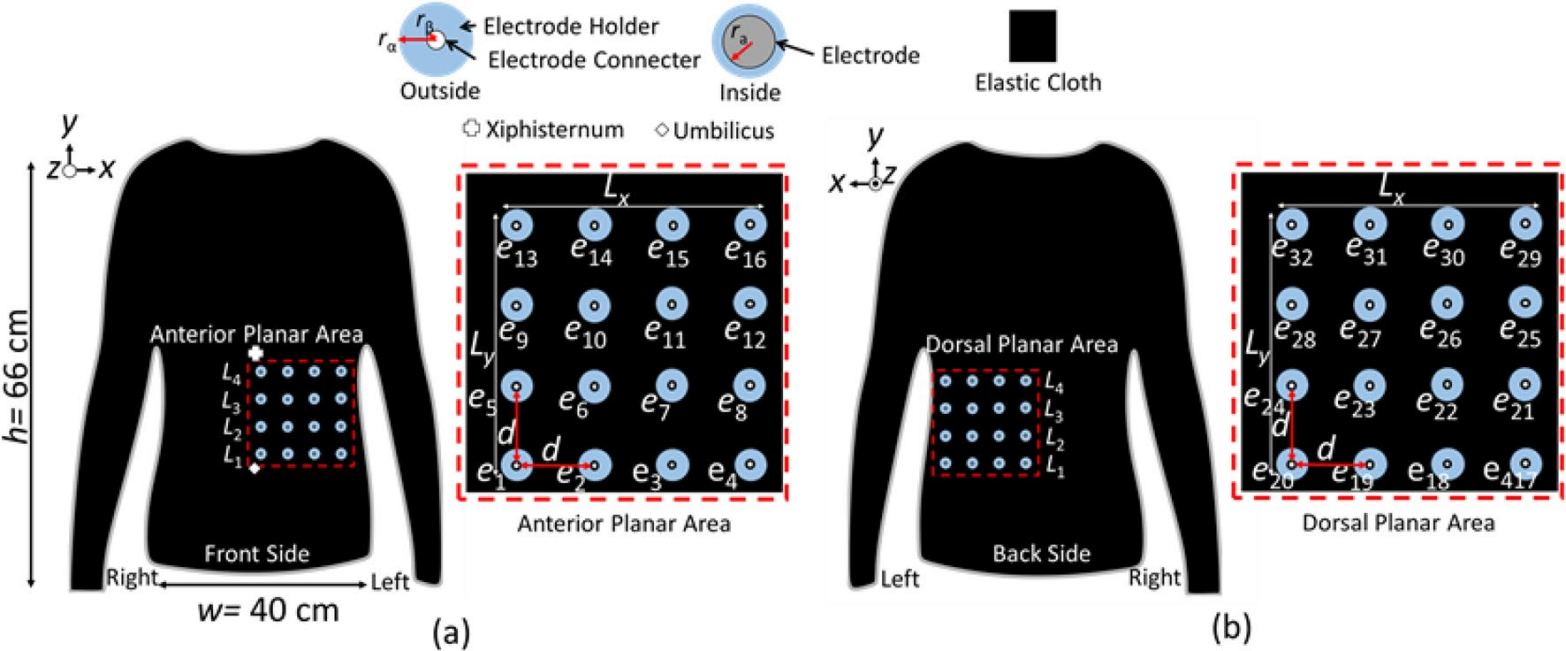
Concept of *g*EIT suit (**a**). Anterior planar area (**b**) Dorsal planar area.

Figure 1 shows the concept of *g*EIT Suit to visualize gastric conductivity distribution inside abdomen. *g*EIT Suit has a function to measure impedance Z by using *E* (*E* = 32 as an example in the Fig. 1) number of electrodes *e*_e_ along the abdomen surface. The *g*EIT Suit consist of elastic cloth (black colour), electrodes holder (blue colour), electrodes connector (white colour) and electrodes (grey colour). The elastic cloth is composed of 88% polyester and 12% polyurethane material with initial width *w* = 40 [cm] and height *h* = 66 [cm], when the elastic cloth is stretched, the maximum width w_max_ = 56 and maximum height *h*_max_ = 88 cm. The electrodes *e*_1_,*e*_2_,…, *e*_*e*_,…, *e*_*E*_(*E* = 32 in the figure as an example) with radius *r*_*e*_ = 8 [mm] are attached across the elastic cloth on the abdomen surface to measure the Impedance Z. The electrodes are covered with electrode holder α with radius *r*_α_ = 1 [cm] made by Poly Lactic Acid (PLA) material to fix the electrode position on elastic cloth surface. Furthermore, the electrode connector β with radius *r*_α_ = 2 [mm] is attached on the centre of electrode holder to connect the electrodes with compact *g*EIT DAQ cables.

The electrodes are arranged according to dual planar configuration as shown in Fig. 1 consist of anterior planar area Fig. 1a in the front side of *g*EIT Suit and dorsal planar area on back side of *g*EIT Suit Fig. 1b with horizontal area length *L*_*x*_ = 17 [cm] and vertical area length *L*_*y*_ = 17 [cm]. Each planar consists of 16 electrodes.

### Compact data acquisition (DAQ) system

**Figure 2:**
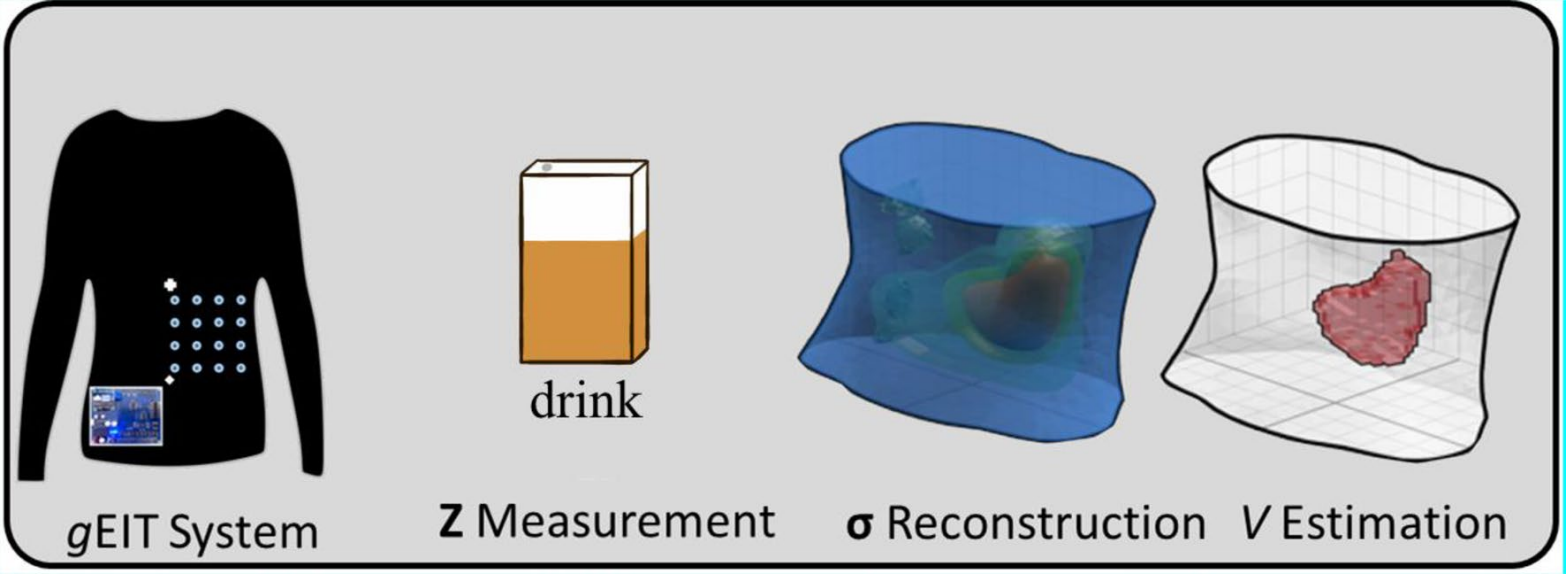
Overview of gastric content volume *V* estimation by *g*EIT Suit with dual-step fuzzy clustering.

**Figure 3:**
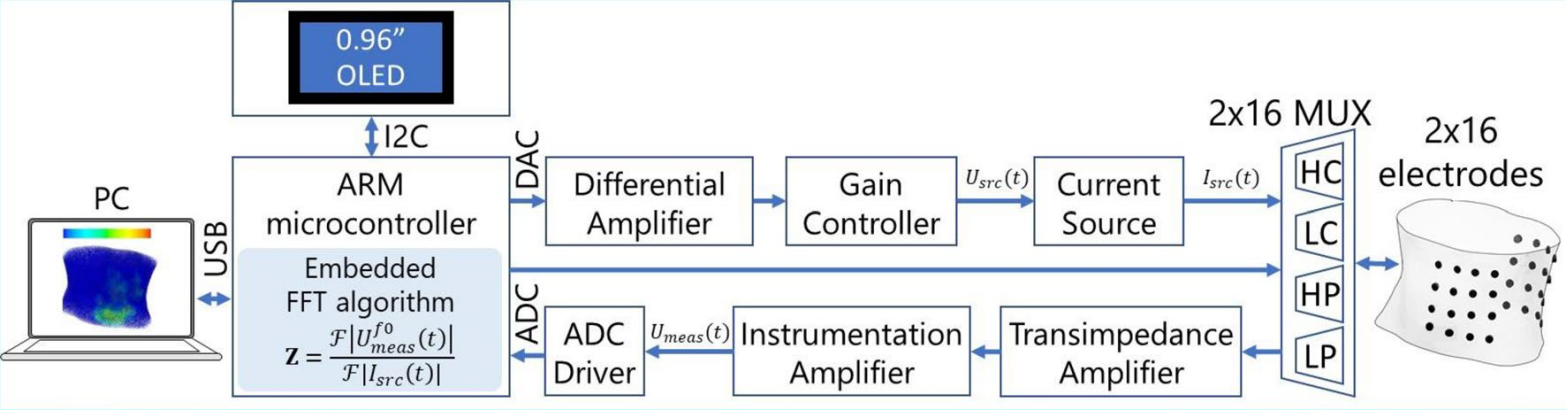
The block diagram of *g*EIT hardware.

Figure 2 shows the compact *g*EIT DAQ system hardware which is connected to elastic suit through electrodes. Further, Fig. 3 shows the block diagram of *g*EIT hardware. The compact *g*EIT DAQ consists of advanced RISC (reduced instruction set computer) machine (ARM) microcontroller, differential amplifier, gain controller, current source, multiplexers (MUX), trans-impedance amplifier, instrumentation amplifier, and ADC driver. The main controller of compact *g*EIT DAQ is ARM Cortex-M4-based microcontroller with 180 MHz clock speed. The compact *g*EIT DAQ has capability to generate the digitized sinusoidal signal through a 10-bit analogue to digital converter (DAC) which then converted to analogue voltage source ${{\varvec{U}}}_{{\varvec{s}}{\varvec{r}}{\varvec{c}}}\left({\varvec{t}}\right)$ by differential amplifier and gain controller. In order to perform the constant current injection, ${{\varvec{U}}}_{{\varvec{s}}{\varvec{r}}{\varvec{c}}}\left({\varvec{t}}\right)$ is converted into current source ${{\varvec{I}}}_{{\varvec{s}}{\varvec{r}}{\varvec{c}}}\left({\varvec{t}}\right)$ by general Howland current circuit. The ${{\varvec{I}}}_{{\varvec{s}}{\varvec{r}}{\varvec{c}}}\left({\varvec{t}}\right)$ injection and voltage measurement ${{\varvec{U}}}_{{\varvec{m}}{\varvec{e}}{\varvec{a}}{\varvec{s}}}\left({\varvec{t}}\right)$ operation is handled by 2 × 16 analogue multiplexers (MUX), which are divided into four section: high current (HC), low current (LC), high potential (HP), and low potential (LP). The ARM microcontroller controls the injection and measurement pattern^15^.

The compact *g*EIT DAQ measured voltage at *t*-th time ${U}_{meas}\left(t\right)$ is processed by trans-impedance and instrumentation amplifier converted into acceptable ARM microcontroller voltage level by analog to digital converter (ADC) driver. The real-time measurement of ${U}_{meas}\left(t\right)$ is measured by the 12-bit ADC of the controller. Then, the $\mathbf{Z}$ measurement is performed by conducting an embedded fast Fourier transform (FFT) algorithm which expressed as,

1 $${\mathbf{Z}} = \frac{{{\mathcal{F}}\left| {U_{meas}^{f0} \left( t \right)} \right|}}{{{\mathcal{F}}\left| {I_{src} \left( t \right)} \right|}}$$

where $\mathcal{F}$ is the Fourier transform of given fundamental ${{\varvec{U}}}_{{\varvec{m}}{\varvec{e}}{\varvec{a}}{\varvec{s}}}^{\boldsymbol{ }}({\varvec{t}})$ signal ${{\varvec{U}}}_{{\varvec{m}}{\varvec{e}}{\varvec{a}}{\varvec{s}}}^{{\varvec{f}}0}({\varvec{t}})$ at the time domain^19^. The measured $\mathbf{Z}$ then sent into PC through USB connection by universal asynchronous receiver-transmitter (UART) at 1,000,000 bps. The specification of *g*EIT hardware is summarized in Table 2^19^.

**Table 2:** Specification of hardware.

Parameter	Value
Measurement speed	10 fps
Frequency	100 Hz ~ 200 kHz
Current injection	1 mA (constant)
Digital signal processing (DSP)	Embedded FFT algorithm
UART baud rate	1,000,000 bps
I/O channel	2 × 16 channels
Switching mode	Full 3D Injection

### Estimation of gastric function by dual-step fuzzy clustering based on reconstructed images

In this stage, the conductivity distribution **σ** is clustered using the dual-step fuzzy clustering into three clusters ^*k*^**σ** (*k* = 3) which are gastric content ^1^**σ**, gastric wall ^2^**σ**, and other abdomen organs ^3^**σ**^18^. The objective of the first step of fuzzy clustering is to eliminate noise conductivity from abdomen organs can be summarized using the following equations:

2 $${}_{{}}^{k} {\mathbf{r}}_{{\text{c}}}^{{\text{j}}} = \frac{{\mathop \sum \nolimits_{nk = 1}^{nk = Nk} {}_{{}}^{k} \sigma_{n} {\varvec{r}}_{n} }}{{\mathop \sum \nolimits_{nk = 1}^{nk = Nk} {}_{{}}^{k} \sigma_{n} }}$$

3 $${}_{{}}^{k} {\mathbf{f}}_{{\text{c}}}^{{\text{j}}} = \frac{{\mathop \sum \nolimits_{n = 1}^{{n_{k} }} (^{k} \mu_{n}^{j} )^{2} {}_{{}}^{k} \sigma_{n} {\varvec{r}}_{n} }}{{\mathop \sum \nolimits_{n = 1}^{{n_{k} }} (^{k} \mu_{n}^{j} )^{2} }}$$

4 $${}_{{}}^{k} \mu_{n}^{j} = 1{\bigr{/}}\mathop \sum \limits_{l = 1}^{l = k} \left( {\frac{{{||\mathbf{r}}_{{\text{n}}} - {}_{{}}^{k} {\mathbf{r}}_{c}^{j}||}}{{{||\mathbf{r}}_{{\text{n}}} - {}_{{}}^{l} {\mathbf{r}}_{c}^{j}||}}} \right)^{2}$$

5 $$^{k} {{\varvec{\upsigma}}} = \arg \min \sum\limits_{k = 1}^{k = K} {\sum\limits_{n = 1}^{N} {\left( {^{k} \mu_{n}^{j} } \right)^{2} {||\mathbf{r}}_{n} - {^{k}\mathbf{f}}_{c}^{{j }} }|| ^{2}}$$

6 $${}_{{}}^{k} {\mathbf{f}}_{c}^{j + 1} - {}_{{}}^{k} {\mathbf{f}}_{c}^{j} = 0$$

where ^*k*^**r**_*c*_^*j*^ in (2) is *k*-th clustered centroid position at *j*-th iteration number, ^*k*^σ_*n*_ is *k*-th clustered conductivity distribution at *n*-th elements, **r**_n_ = [*x*_n_ , *y*_n_ , *z*_n_] is *n*-th mesh element position vector, *n*_k_ (1 ≤ *n*_k_ ≤ *N*_k_ ) is the *n*-th mesh which belongs to ^*k*^**σ**. At fuzzy centroid calculation process, fuzzy centroid position of *k*-th cluster ^*k*^**f**_c_ from the *n*-th mesh element position vector ^*k*^**r**_*n*_ is calculated by (3) and (4). Then, the elements of ^*k*^**σ**_*n*_ are relocated into a new clustered conductivity distribution by calculating Euclidean distance between the position of **r**_*n*_ and ^*k*^**f**_*c*_ by (5). The final clustered conductivity distribution is achieved while the difference between ^*k*^**r**_*n*_^*j*^ and ^*k*^**r**_*n*_^*j*+1^ equal to 0 as shown in Eq. (6). Finally, the second dual-step fuzzy clustering is started by extracting the only gastric conductivity distribution *σ by eliminating the conductivity noise from abdomen organs ^3^**σ** using following equation

7 $${}_{{}}^{*} \sigma_{n} = \left\{ {\begin{array}{*{20}c} {0,{\upsigma }_{n} \in {}_{{}}^{3} {{\varvec{\upsigma}}}} \\ {{\upsigma }_{n} ,{\upsigma }_{n} \notin {}_{{}}^{3} {{\varvec{\upsigma}}}} \\ \end{array} } \right.$$

## Experiment setup and protocol

### Experimental setup

**Figure 4:**
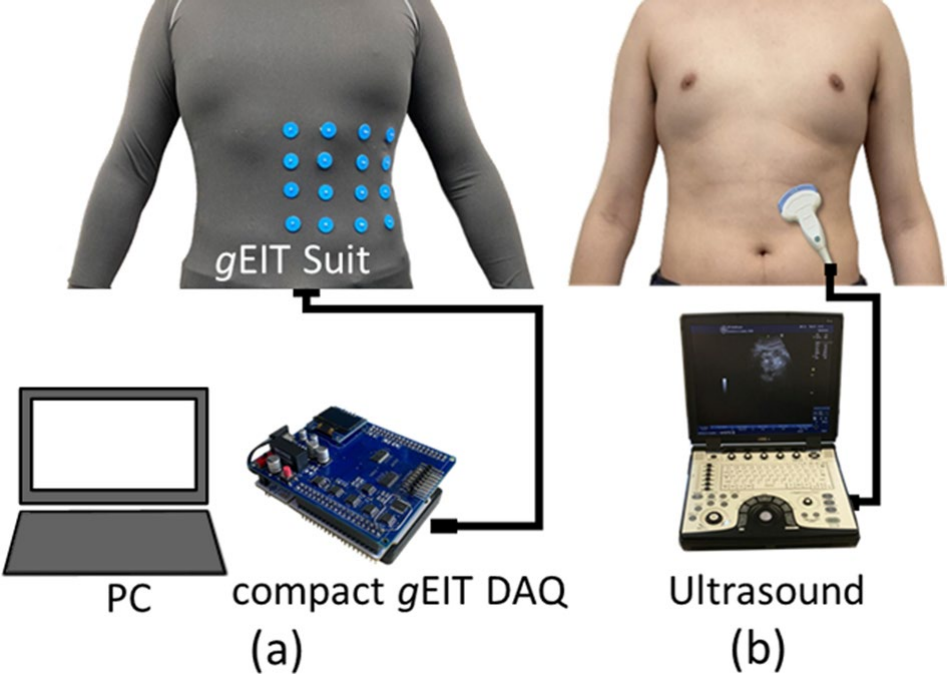
Experimental Setup (**a**) *g*EIT system experimental setup, (**b**) ultrasound experimental setup.

Figure 4 shows the experimental setup consisting of a) *g*EIT Suit composed of elastic suit fabricated in the stage 2A, a compact *g*EIT DAQ developed in the stage 2B and a Personal Computer (Mouse Computer LM-iH800) with 3.2 GHz Intel Core i7-8700 and 64 Gb RAM was used in the reconstruction of gastric conductivity distribution by *g*EIT Suit and estimate the gastric volume *V* by Fuzzy Clustering.

### Experimental protocol

**Figure 5:**
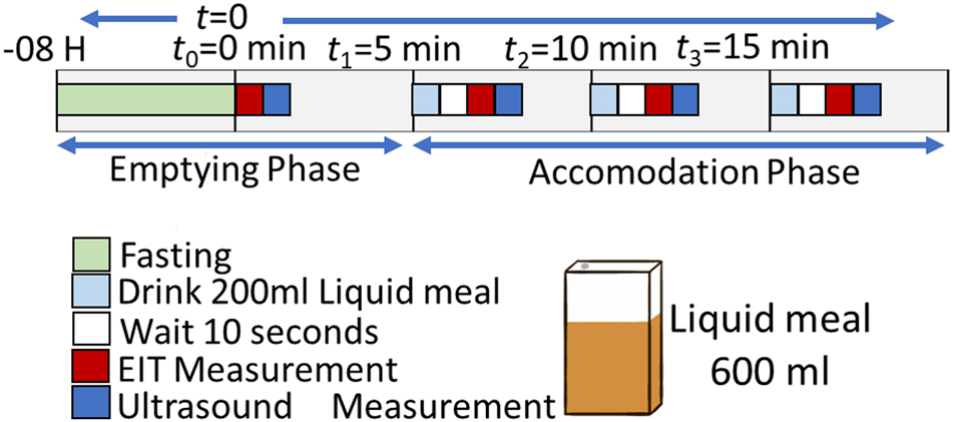
Experimental protocol.

Eight healthy young men (age: 25 ± 5 years, body mass index 21.94 ± 3.27) volunteered for this study. Figure 5 shows the experimental protocol consist of two phases: gastric empty phase and gastric accommodation phases using *g*EIT Suit measurement Fig. 4a and ultrasound measurement system Fig. 4b.

Gastric empty phase is defined as measurement of the gastric area during fasting condition (no meal or drink) for at least 8 h, on the other hand the gastric accommodation phases is defined as the measurement of gastric area using 200 mL, 400 mL and 600 mL liquid meal (Aisokaru 100, Nestle Health Science Japan) Measurement of ultrasound area of the antrum was obtained by abdominal ultrasound probe (frequency 4 MHz) manufactured by GE Healthcare.

In all experiment parts, volunteered subjects were measured in a standing position. Chiba University Ethic Research Committee approved this protocol and all volunteers gave informed written consent before experiments. All study procedures were conducted in accordance with the Declaration of Helsinki. The subjects were asked to fast for at least 8 Hours and avoid to drink alcohol, caffeine and any medication that could affect their gastric function in the gastric emptying phase. The subjects underwent the gastric emptying scan at 8 h + 0 min (*t*_0_) followed by *g*EIT Suit measurement and ultrasound measurement. After that the gastric accommodation start at 8 h + 5 min (*t*_1_) by drinking 200 mL of liquid drink followed by *g*EIT Suit and US measurements. Next in the 8 h + 10 min (*t*_2_), the subject were asked to drink 200 mL of liquid meal followed by *g*EIT Suit and US measurements. Lastly, in the 8 h + 15 min (*t*_3_), the subject were asked to drink 200 mL of liquid meal followed by *g*EIT Suit and US measurements.

### Image reconstruction method

**Figure 6:**
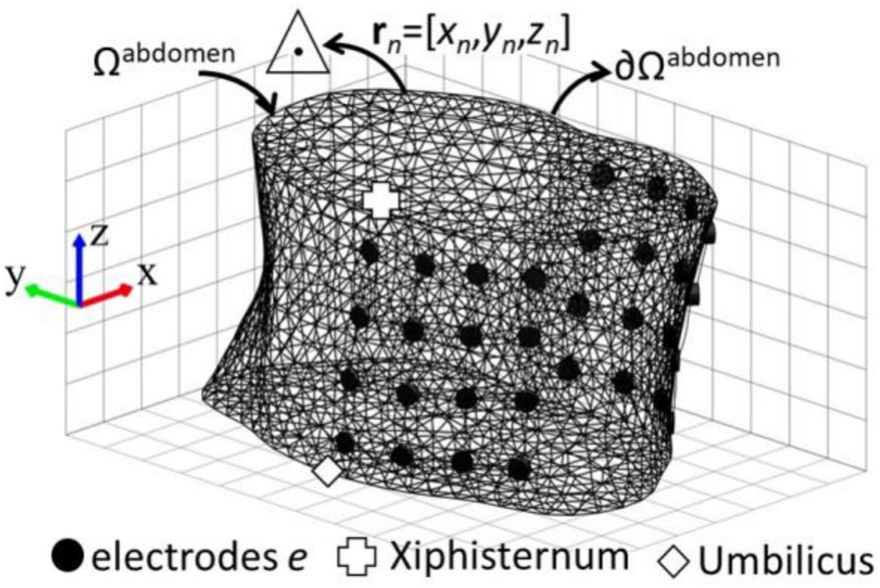
3-dimensional mesh inside abdomen *Ω*^abdomen^.

Figure 6 shows the structure of *g*EIT Suit around an abdomen boundary ∂Ω_abdomen_ with electrodes number *e* (1 ≤ *e* ≤ *E*, *E* = 32 = 8 electrodes per one layer × 4 layers as an example in Fig. 6. The abdomen inside is discretised into volumetric elements with number *N* as shown in Fig. 6. In order to obtain conductivity distribution by *g*EIT, time difference (td-*g*EIT) Gauss–Newton image reconstruction is used using following equation

8 $${{\varvec{\upsigma}}}^{j + 1} = {{\varvec{\upsigma}}}^{j} - \left( {{\mathbf{J}}^{{\text{T}}} {\mathbf{J}} + \lambda {\mathbf{R}}} \right)^{ - 1} {\mathbf{J}}^{{\text{T}}} {\Delta }{\mathbf{Z}}$$

where **R** is a regularization matrix, and *λ* is a relaxation factor scalar which is automatically determined by L-Curve method^20^, Δ**Z** = [Δ*Z*_1_, …, Δ*Z*_*m*_, …, Δ*Z*_*M*_]^T^ $\in {\mathbb{R}}^{M}$ is the normalized measured impedance between the impedance at initial time *t*_0_ and measurement time *t* which is written by

9 $${\Delta }Z_{m} \left( {t - t_{0} } \right) = \frac{{Z_{m} \left( t \right) - Z_{m} \left( {t_{0} } \right)}}{{Z_{m} \left( {t_{0} } \right)}}$$

where m is measured voltage pattern and M is total number of measurement patterns.

## Experimental results and discussion

### Gastric impedance

**Figure 7:**
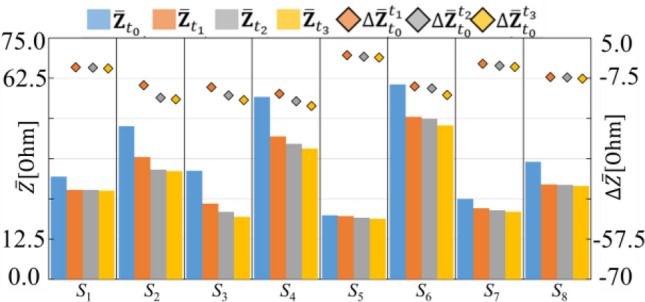
Comparison of average measured impedance $\overline{\mathbf{Z} }$, delta measured impedance $\overline{\Delta \mathbf{Z} }$.

Based on the impedance measurement in chapter 2, Fig. 7 shows average measured impedance and delta measured impedance. The blue bar chart in the Fig. 7 indicates the average impedance during empty stomach at *t*_0_ ${(\overline{\mathbf{Z}} }_{{{\varvec{t}}}_{0}})$ . On the other hand, the orange, grey and yellow bar chart represents the average impedance during the accommodation phase when the subjects were drinking 200 mL, 400 mL and 600 mL of a liquid meal at *t*_1_ ${(\overline{\mathbf{Z}} }_{{{\varvec{t}}}_{1}})$, *t*_2_ ${(\overline{\mathbf{Z}} }_{{{\varvec{t}}}_{2}})$, and *t*_3_ ${(\overline{\mathbf{Z}} }_{{{\varvec{t}}}_{3}})$ respectively. According to the Fig. 7, the measured impedance the during the empty phase ${\overline{\mathbf{Z}} }_{{{\varvec{t}}}_{0}}$ has the highest value as compared with the average impedance during the accommodation phase (${\overline{\mathbf{Z}} }_{{{\varvec{t}}}_{1}}{,\overline{\mathbf{Z}} }_{{{\varvec{t}}}_{2}},{\overline{\mathbf{Z}} }_{{{\varvec{t}}}_{3}}$). Furthermore, during the accommodation phase, the $\overline{\mathbf{Z} }$ were gradually decrease at *t*_1_, *t*_2_, *t*_3_ (${\overline{\mathbf{Z}} }_{{{\varvec{t}}}_{1}}{>\overline{\mathbf{Z}} }_{{{\varvec{t}}}_{2}}>{\overline{\mathbf{Z}} }_{{{\varvec{t}}}_{3}}$). In order to quantitatively evaluate the *g*EIT Suit to estimate gastric content volume, the delta impedance between gastric empty phase and accommodation phases were calculated using following equation :

10 $$\Delta \overline{{{\mathbf{Z}}_{{t_{0} }}^{t} }} = \left( {\sum\limits_{m = 1}^{m = M} {Z_{m} \left( t \right) - Z_{m} \left( {t_{0} } \right)} } \right)/M$$

where *Z*_*m*_(*t*_0_), *Z*_*m*_(*t*_1_) are the impedance measurement at *m*-th measurement pattern during empty and accommodation phase respectively.

The orange, grey and yellow diamond chart in the Fig. 7 represents the delta average impedance during the accommodation phase when the subjects were drinking 200 mL, 400 mL and 600 mL of liquid meal at *t*_1_ ($\Delta {\overline{\mathbf{Z}} }_{{{\varvec{t}}}_{0}}^{{{\varvec{t}}}_{1}}$), *t*_2_ ($\Delta {\overline{\mathbf{Z}} }_{{{\varvec{t}}}_{0}}^{{{\varvec{t}}}_{2}}$), and *t*_3_ ($\Delta {\overline{\mathbf{Z}} }_{{{\varvec{t}}}_{0}}^{{{\varvec{t}}}_{3}}$) respectively. By the *g*EIT Suit measurement, the average value of delta impedance is $\overline{\Delta \mathbf{Z} }$ = − 9.27 [Ohm].

### Gastric conductivity distribution

**Figure 8:**
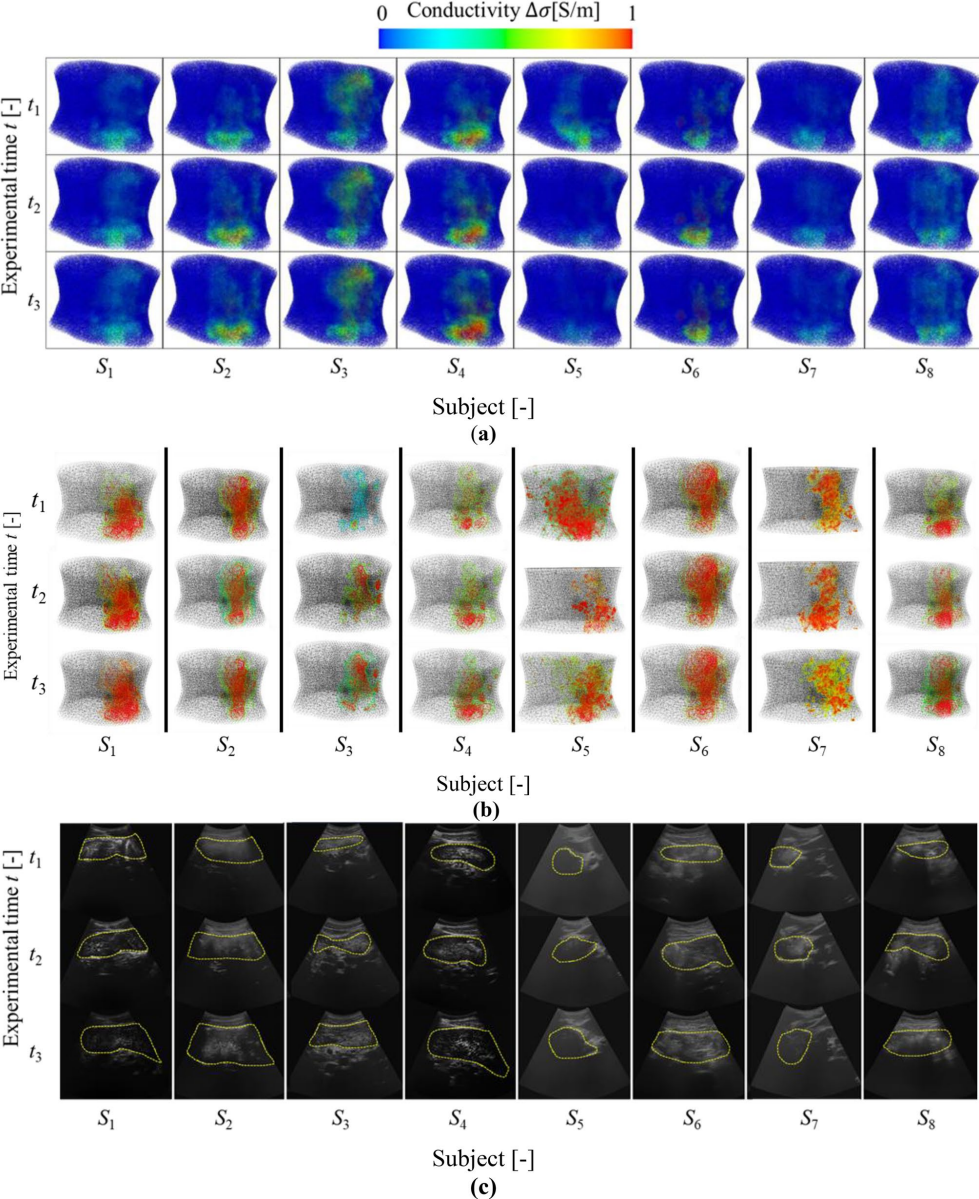
(**a**) Visualization of gastric conductivity distribution **σ** by *g*EIT Suit, (**b**) Extraction of clustered gastric conductivity distribution ^*k*^**σ** by fuzzy clustering, (**c**) Evaluation of gastric area by ultrasound.

Based on the reconstruction of gastric conductivity distribution **σ** by using *g*EIT Suit in the chapter 2. Figure 8a show the comparison of **σ**. Based on the Fig. 8a the **σ** are well visualized.

In order to quantitatively evaluate the performance of *g*EIT Suit to measure gastric content volume, the spatial mean conductivity $\langle {\varvec{\upsigma}}\rangle$ were calculated using following equation:

11 $$\left\langle {{\varvec{\upsigma}}} \right\rangle_{t} = \left( {\sum\limits_{n = 1}^{n = N} {\sigma_{n}^{t} } } \right)/N$$

${{\varvec{\sigma}}}_{{\varvec{n}}}^{{\varvec{t}}}$ is the conductivity distribution in *n*-th mesh voxel at *t* accommodation phases.

**Figure 9:**
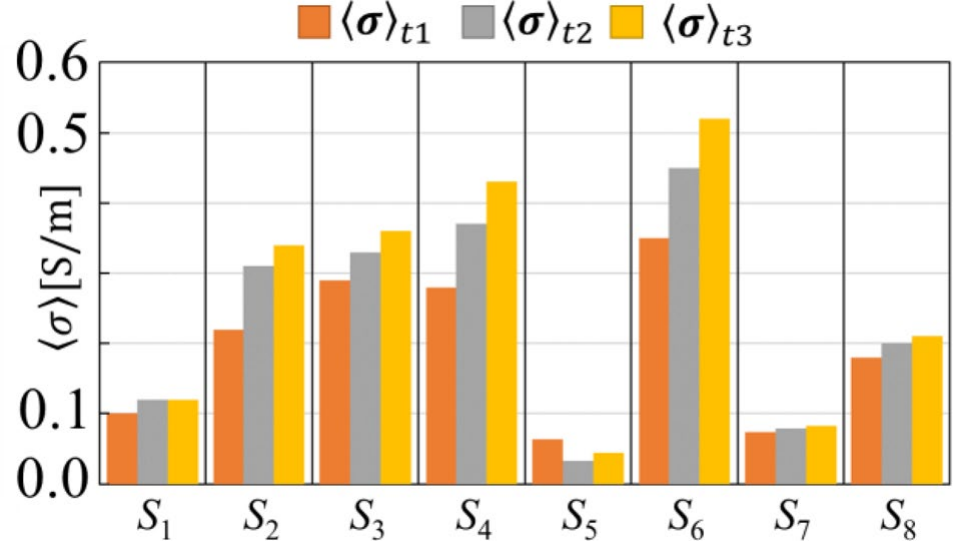
Spatial mean of gastric conductivity $\langle {\varvec{\upsigma}}\rangle$ distribution.

Figure 9 shows the spatial mean gastric conductivity distribution $\langle {\varvec{\upsigma}}\rangle$ . The orange, grey and yellow bar chart represents the $\langle {\varvec{\upsigma}}\rangle$ during the accommodation phase when the subjects were drinking 200 mL, 400 mL and 600 mL of liquid meal at *t*_1_ $({\langle {\varvec{\upsigma}}\rangle }_{{{\varvec{t}}}_{1}})$, *t*_2_ $({\langle {\varvec{\upsigma}}\rangle }_{{{\varvec{t}}}_{2}})$, and *t*_3_ $({\langle {\varvec{\upsigma}}\rangle }_{{{\varvec{t}}}_{3}})$, respectively. Based on the Fig. 9, the average value of $\langle {\varvec{\upsigma}}\rangle$=0.23 [S/m].

### Gastric Accommodation Volume

Based on the clustered gastric conductivity distribution ^*k*^**σ** by using dual step fuzzy clustering in the chapter 2, Fig. 8b shows comparison of clustered gastric conductivity distribution between healthy subjects. According to the Fig. 8b the gastric volume are qualitatively visualized during the accommodation phases when the subjects were drinking 200 mL, 400 mL and 600 mL of liquid meal at *t*_1 (_^*k*^ ${{\varvec{\upsigma}}}_{{{\varvec{t}}}_{1}}$), _(_^*k*^ ${{\varvec{\upsigma}}}_{{{\varvec{t}}}_{2}}$), and _(_^*k*^ ${{\varvec{\upsigma}}}_{{{\varvec{t}}}_{3}}$)respectively.

**Figure 10:**
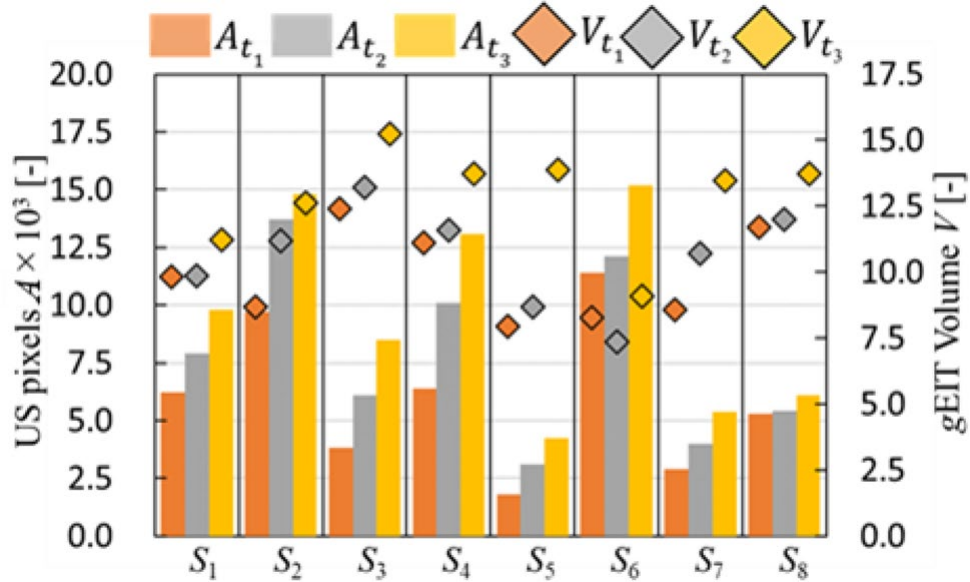
Comparison of gastric volume from *g*EIT suit *V*_*t*_ and gastric area from ultrasound *A*_*t*_.

**Figure 11:**
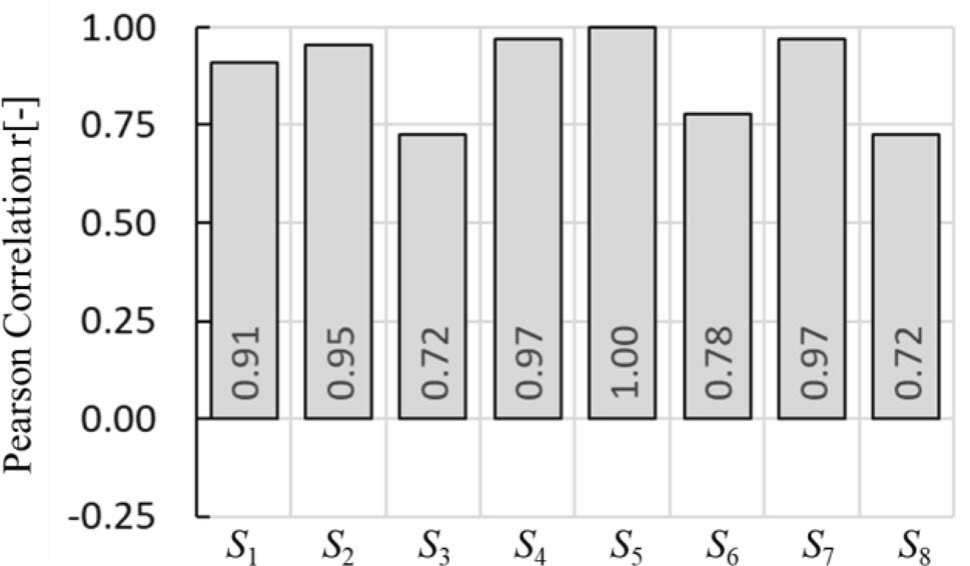
Comparison of Pearson correlation *r.*

In order to quantitatively evaluate the performance of *g*EIT Suit to measure gastric content volume, the gastric volume *V*_*t*_ is defined using following equation:

12 $$V_{t} = \left( {\left| {{}_{{}}^{k} {\varvec{\sigma}}_{t} } \right|/N} \right) \times 100\%$$

where |^*k*^ ${{\varvec{\upsigma}}}_{{\varvec{t}}}$| is the number of voxel in the clustered gastric conductivity at *t*-th time, *N* is total number of mesh inside the *g*EIT Suit boundary. Figure 10 shows the gastric volume from *g*EIT Suit between subjects and also gastric area from ultrasound measurement. Furthermore, Fig. 8c shows the comparison of gastric ultrasound images of healthy subjects. In order to quantitatively evaluate the gastric ultrasound images, the gastric area *A*_*t*_ is defined as the total pixels inside the gastric boundary (dashed yellow line) in the ultrasound images. The Pearson correlation *r* is defined using the following equation

13 $$r = \frac{1}{T}\mathop \sum \limits_{t = 1}^{t = T} \frac{{\left[ {\left( {V_{t} - \overline{\user2{V}}} \right)\left( {A_{t} - \overline{\user2{A}}} \right)} \right]}}{{\sqrt {\left[ {\left( {V_{t} - \overline{\user2{V}}} \right)^{2} - \left( {A_{t} - \overline{\user2{A}}} \right)^{2} } \right]} }} \left[ - \right]$$

where *V*_*t*_ is gastric volume from *g*EIT Suit at experimental condition *t*-th time. $\overline{{\varvec{V}}}$ is mean of gastric volume by *g*EIT Suit. *A*_*t*_ is defined as the pixels inside the gastric boundary in the ultrasound images at experimental condition *t*-th time. $\overline{{\varvec{A}}}$ is mean of *At*. and so on Where *T* = 3 is total number of measurements during the gastric accommodation phases. Figure 11 shows the comparison of Pearson correlation *r* among healthy subjects. Based on the figure, *g*EIT suit has good agreement with ultrasound images with average *r* = 0.88[–].

In order to quantitative evaluation of *g*EIT Suit measurement, *t*-test was also applied that evaluates the probability distribution of a hypothesis as shown in following equations:

14 $$\tau \left( Z \right) = \frac{{\overline{{\Delta Z_{t - t0} }} \sqrt S }}{{\sqrt {\frac{1}{S}\mathop \sum \nolimits_{s = 1}^{S} \left( {\left( {Z_{t}^{s} - Z_{t0}^{s} } \right) - \left( {\overline{{\Delta Z_{t - t0} }} } \right)} \right)^{2} } }}$$

15 $$\tau \left( \sigma \right) = \frac{{\overline{{\Delta \sigma_{t - t0} }} \sqrt S }}{{\sqrt {\frac{1}{S}\mathop \sum \nolimits_{s = 1}^{S} \left( {\left( {\sigma_{t}^{s} - \sigma_{t0}^{s} } \right) - \left( {\overline{{\Delta \sigma_{t - t0} }} } \right)} \right)^{2} } }}$$

16 $$\tau \left( V \right) = \frac{{\overline{{\Delta V_{t - t0} }} \sqrt S }}{{\sqrt {\frac{1}{S}\mathop \sum \nolimits_{s = 1}^{S} \left( {\left( {V_{t}^{s} - V_{t0}^{s} } \right) - \left( {\overline{{\Delta V_{t - t0} }} } \right)} \right)^{2} } }}$$

where $\tau$ is the *t*-test value of the mean impedance *Z*_*t*_, spatial mean conductivity *σ*_t_, gastric volume *V*_*t*_, *S is* $s$ ubject number. ${Z}_{t}^{s}$ is mean measured impedance by *g*EIT Suit at subject number *s* and *t*-th experimental condition, ${\sigma }_{t}^{s}$, ${V}_{t}^{s}$ are same as spatial mean conductivity and gastric volume by *g*EIT Suit. $\overline{\Delta {Z }_{t-t0}}$ is mean of difference between experimental condition *t*, *t*_0_ as $\overline{\Delta {Z }_{t-t0}}=\frac{1}{S}\sum_{s=1}^{S}({Z}_{t}^{s}-{Z}_{t0}^{s})$. $\overline{\Delta {\sigma }_{t-t0}}$, $\overline{\Delta {V }_{t-t0}}$ are same at spatial mean conductivity and gastric volume. Finally, the probability value (*p*-value) *p*(*Z*), *p*(*σ*), *p*(*V*), are obtained by τ(*Z*), τ(*σ*), τ(*V*).

Table 3 shows the result, the *p*-values obtained by t-test. *p*(*Z*) and *p*(*σ*) is extremely smaller than significance level 0.10 and *p*(*V*) also smaller than that. So, null hypothesis are rejected. It can be say there is a significant difference between those mean value of *Z*, *σ*, *V* among experimental condition by *g*EIT Suit measurement.

**Table 3:** Result of paired samples *t*-test for $\overline{\mathrm{Z} }$(*t*_3_–*t*_0_), $\langle \upsigma \rangle$(*t*_3_–*t*_1_), *V*(*t*_3_–*t*_1_).

Items	$\overline{\mathrm{Z} }$ (*t*_3_,*t*_0_)		〈*σ*〉(*t*_1_,*t*_3_)		*V*(*t*_1_,*t*_3_)	
	*t*_3_	*t*_0_	*t*_1_	*t*_3_	*t*_1_	*t*_3_
Mean	29.7262	39.0041	11.8084	12.6070	0.1947	0.2634
SD	9.7906	13.6820	1.7405	1.9900	0.1013	0.1631
SE	3.4615	4.8373	0.6153	0.7036	0.0358	0.0577
Mean Diff	− 9.2779		− 0.7986		− 0.0687	
*t*_0.025_	2.1790					
*t*-value	− 4.5927		− 2.7643		− 1.7939	
*p*-value	0.0013		0.0140		0.0664	

## Conclusions

Gastric functional monitoring has been proposed by Gastric Electrical Impedance Tomography (*g*EIT) Suit. The proposed method has 4 stages: (1) Fabrication of *g*EIT Suit by integration of elastic suit with dual-planar EIT electrode arrangement, (2) Measurement of gastric impedance **Z** by using compact *g*EIT data acquisition system (DAQ), (3) Reconstruction of gastric conductivity distribution **σ** by using time-difference gauss–newton algorithm and 4) Estimation of gastric volume *V* by using dual-step fuzzy clustering. The key finding of this research are :

1) By *g*EIT Suit measurement, the subjects have a value of temporal mean impedance $\overline{\Delta {\varvec{Z}} }$ = − 9.27 [Ohm], spatial mean conductivity distribution $\langle {\varvec{\sigma}}\rangle$=0.23[–], and mean gastric volume $\overline{V }$= 12.60[%].

2) The *g*EIT Suit statistically accurate with high pearson correlation mean value *r*=0.88[–] and low *p*-value *p*(*Z*)=0.0013, *p*(*σ*)=0.0140, *p*(*V*)=0.0664[–].

## Data Availability

The datasets used and/or analyzed during the current study available from the corresponding author on reasonable request. According to Bioethics Committee in Faculty of Engineering, Chiba University (ethical code R2-02), all subjects gave written informed consent for the study after receiving a detailed explanation for the purposes, potential benefits, and risks associated with participation. All study procedures were conducted in accordance with the Declaration of Helsinki and the research code of ethics of Chiba University and were approved by the Committee for Human Experimentation of Chiba University.
